# Protective Effects of* Broussonetia kazinoki* Siebold Fruit Extract against Palmitate-Induced Lipotoxicity in Mesangial Cells

**DOI:** 10.1155/2019/4509403

**Published:** 2019-01-08

**Authors:** Donghee Kim, Hyo-Jin Kim, Seon-Heui Cha, Hee-Sook Jun

**Affiliations:** ^1^Lee Gil Ya Cancer and Diabetes Institute, Gachon University, Incheon 21999, Republic of Korea; ^2^College of Pharmacy, Gachon University, Incheon 21936, Republic of Korea; ^3^Gachon Medical and Convergence Institute, Gachon Gil Medical Center, Incheon 21565, Republic of Korea

## Abstract

Diabetic nephropathy is one of the most serious complications of diabetes. Lipotoxicity in glomerular mesangial cells is associated with the progression of diabetic nephropathy. Paper mulberry,* Broussonetia kazinoki* Siebold (BK), has been used in oriental medicine for human health problems. However, to date, the beneficial effect of BK fruit has not been studied. In this study, we investigated the protective effect of an ethanolic extract of BK fruit (BKFE) against palmitate- (PA-) induced toxicity in mesangial cells. BKFE significantly increased the viability of PA-treated SV40 MES13 cells. BKFE significantly inhibited PA-induced apoptosis and decreased the expression of apoptotic genes, cleaved caspase-3, and cleaved PARP. Moreover, BKFE inhibited the expression of endoplasmic reticulum (ER) stress-related genes, such as BiP, phosphorylated eIF2*α*, cleaved ATF6, and spliced XBP-1, in PA-treated SV40 MES13 cells. BKFE decreased PA-induced ROS production. In addition, BKFE activated the transcription factor Nrf2 and increased the expression of antioxidant enzymes. However, knockdown of Nrf2 using siRNA suppressed this BKFE-induced increase in antioxidant enzyme expression. Furthermore, the protective effect of BKFE on PA-induced apoptosis was significantly reduced by Nrf2 knockdown. In conclusion, BKFE induced the expression of antioxidant enzymes via activation of Nrf2 and protected against PA-induced lipotoxicity in mesangial cells.

## 1. Introduction

Diabetic nephropathy (DN) is one of the most serious complications in patients with diabetes mellitus [1]. One important feature of DN is the gradual, inexorable scarring of the renal glomerulus, known as glomerulosclerosis [2]. Glomerulosclerosis in DN is caused by accumulation of extracellular matrix (ECM) proteins in the mesangial interstitial space, which results in fibrosis [3]. Mesangial cells are major components of the glomerular mesangium and primary targets of diabetes; thus, they are intimately involved in glomerulosclerosis [4]. In response to diabetic factors, such as hyperglycemia, oxidative stress, and excess free fatty acids (FFAs), mesangial cells acquire an activated phenotype and then undergo hypertrophy and proliferation or undergo apoptosis, leading to kidney dysfunction [4, 5]. Lipid toxicity is an important mechanism in DN, and mesangial cells are susceptible to lipotoxicity. Thus, lipotoxicity-induced apoptosis of mesangial cells has been implicated in the development of renal failure [6, 7]. Therefore, inhibition of lipotoxicity in mesangial cells is a potential therapeutic approach to prevent and treat DN.

Oxidative stress is involved in the pathology of DN. Saturated FFAs induce intracellular reactive oxygen species (ROS) production, as observed in various cell types, including pancreatic *β*-cells and mesangial cells [6, 8, 9]. Palmitic acid (PA), a saturated FFA, elicits unfolded protein response (UPR) and endoplasmic reticulum (ER) stress. ER stress is a physiological and pathological response to excessive accumulation of unfolded or misfolded proteins in the ER. ER stress has been implicated in lipotoxicity-induced apoptosis in multiple cell types, including renal cells [5, 10, 11]. Therefore, antioxidants may exert a protective effect in mesangial cells under palmitate-induced lipotoxic conditions.

Natural plants have been regarded as an important source of therapeutic agents for human health problems, including diabetes [12–15]. The twigs and root and stem barks of* Broussonetia kazinoki* Siebold (Moraceae), which we thereafter refer to as BK, have been used in oriental medicine to ameliorate vision, inflammatory, and infectious diseases. The extracts and bioactive phytochemicals of these materials have been reported to have anti-inflammatory, antidiabetic, antihyperglycemic, and anticancer effects [16–19]. The fruit of BK, known as “Jeo-sil-ja” in Korea, has also been used in oriental medicine. It is known to relieve back pain, neuralgia, diuretic action, and swelling, as well as heal dermatitis and restore kidney functions [20]. However, the beneficial effect of BK fruit has not been reported. Thus, we investigated the protective effect of an ethanolic extract of BK fruit (BKFE) against palmitate-induced lipotoxicity in mesangial cells, as well as the mechanisms involved in the antilipotoxic effect of BKFE.

## 2. Materials and Methods

### 2.1. Preparation of BKFE

Dried BK fruits were purchased from an oriental drug store (Kwang Myung Dang Co., Ulsan, Korea), homogenized using a grinder, and then extracted with 80% ethanol. The extract was evaporated* in vacuo* and dissolved in dimethyl sulfoxide (DMSO; Duchefa Biochemie B.V., Haarlem, Netherlands) to a concentration of 50 mg/ml, and then further diluted with a culture medium to the required concentration.

### 2.2. Palmitate Preparation

A stock solution of PA (Sigma, St. Louis, MO, USA) was prepared by conjugating PA with fatty acid-free bovine serum albumin (FAF-BSA, Sigma), as reported previously [21]. In brief, PA was dissolved in Dulbecco's Phosphate-Buffered Saline (DPBS; Welgene Inc., Daegu, Korea) at 60°C for 20 min to make a 20 mM stock solution, and the pH was adjusted to 7.0~7.4 with 1 M NaOH. FAF-BSA was dissolved in DPBS. Next, 20 mM PA solution was diluted in 5% FAF-BSA solution at a ratio of 1:3 (v/v) to generate a 5 mM PA stock solution. Next, PA was diluted in a culture medium to make a 100 *μ*M PA working solution. An 0.08% FAF-BSA solution was used as a control.

### 2.3. Cell Culture

SV40-transformed murine glomerular mesangial (SV40 MES13) cells were obtained from American Type Culture Collection (ATCC, Rockville, MD, USA) and maintained in a mixture of DMEM and F-12 medium (3:1) containing 5% fetal bovine serum, 100 U/mL penicillin, 100 *μ*g/mL streptomycin, and 14 mM HEPES at 37°C in an atmosphere containing 5% CO_2_ and 95% air. In all experiments except the cell viability assay, the cells were incubated in 20 *μ*g/ml BKFE prior to treatment with 100 *μ*M PA. DMSO and FAF-BSA were used as vehicles for BKFE and PA, respectively.

### 2.4. Cell Viability Assay

Cell viability was measured by the water-soluble tetrazolium salt-1 (WST-1) assay. Briefly, SV40 MES13 cells (1×10^4^ cells/well) were seeded on 96-well plates. After 16-18 h culture, the cells were treated with BKFE and/or PA. The cells were treated with different concentrations of PA (12.5-400 *μ*M) for 24 h for cell viability and BKFE (0.5-40 *μ*g/ml) for 24 h for toxicity. To determine the protective effects of BKFE, cells were pretreated with vehicle (DMSO, control) or 0.5-40 *μ*g/ml BKFE for 1 h, and subsequently incubated with or without 100 *μ*M PA for 24 h at 37°C. Cell viability was measured by incubating the cells with 10 *μ*l of D-Plus™ CCK reagent (Dongin LS, Seoul, Korea) for 2 h at 37°C. Next, optical density (OD) at 450 nm was determined using a VersaMax Microplate Reader (Molecular Devices, LLC, Sunnyvale, CA, USA). The OD value of control cells was considered to represent 100% viability. For use in the subsequent studies, we chose a PA concentration of 100 *μ*M, which showed a 50% cell viability, and BKFE dose of 20 *μ*g/ml, which showed the highest protective effects.

### 2.5. Annexin V and Propidium Iodide Staining

Apoptosis was detected using an Annexin V fluorescein isothiocyanate (FITC)/propidium iodide (PI) apoptosis detection kit (BD Biosciences, Franklin Lakes, NJ, USA) according to the manufacturer's instruction. Briefly, the cells were plated in 6-well culture plates at a density of 1.5×10^5^ cells/well. After treatment with 20 *μ*g/ml BKFE and/or 100 *μ*M PA for 24 h, the cells were trypsinized and collected together with floating dead cells. The cells were then washed twice with cold DPBS and resuspended in 1× binding buffer. Annexin V-FITC and PI were added to the tube and the cells were incubated for 15 min at room temperature in the dark. Next, the cells were analyzed using flow cytometry (FACS LSR II, BD Biosciences, CA, USA). Annexin V^−^/PI^−^ population was regarded as normal healthy cells. Apoptotic cells were determined by the sum of early and late apoptotic cells and were indicated as a percentage of the total number of cells.

### 2.6. Western Blotting

SV40 MES13 cells (3×10^5^ cells) were seeded on 60-mm dishes, incubated with vehicle (control) or 20 *μ*g/ml BKFE for 1 h, and then further incubated with or without 100 *μ*M PA for 18 h. The cells were lysed in Mammalian Protein Extraction Buffer (GE Healthcare, Milwaukee, WI, USA) containing a protease and phosphatase inhibitor cocktail. Total proteins were extracted and quantified using the BCA assay. Approximately 20-50 *μ*g of the lysates was electrophoresed and transferred to polyvinylidene difluoride membranes (PVDF, Millipore, Billerica, MA, USA). The membranes were incubated with 5% skimmed milk for 1 h at room temperature, and then incubated with primary antibodies overnight at 4°C. The following antibodies were used at the indicated dilution: 1:2000 (anti-Caspase-3, anti-PARP, anti-BiP, anti-eIF2*α*, anti-p-eIF2*α* and anti-Catalase; Cell Signaling Technology, Boston, MA, USA); 1:1000 (anti-Nrf2, anti-HO-1; Abcam, Cambridge, MA, USA); 1:2000 (anti-*β*-actin and anti-Lamin B; Santa Cruz Biotechnology, Santa Cruz, CA, USA). After washing, the membranes were incubated with secondary antibodies conjugated with horseradish peroxidase (Santa Cruz Biotechnology; Jackson Immunoresearch, West Grove, PA, USA) for 1 h at room temperature. Signals were detected using WESTSAVE (Ab Frontier, Seoul, Korea) and visualized using a LAS-4000 mini system (Fujifilm Corp., Tokyo, Japan). The ImageJ software (National Institute of Health, Bethesda, MD, USA) was used to quantify the intensity of protein bands.

### 2.7. RNA Extraction and Reverse Transcription-Polymerase Chain Reaction (RT-PCR)

Total RNA was extracted from the cultured cells using RNAiso Plus Reagent (TaKaRa Bio Inc., Shiga, Japan), and cDNA was synthesized from 2 *μ*g of RNA using a PrimeScript™ 1st strand cDNA synthesis kit (TaKaRa Bio Inc.) following the manufacturer's instructions. To evaluate the relative expression levels of XBP-1u/XBP-1s, RT-PCR analysis was performed using PCR SuperMix (Invitrogen). Murine XBP-1 primer sequences were as follows: 5′-GACTGACTGACTGATCGATC-3′, and 5′-GATCGATCGATCGATC-3′. GAPDH was used as a loading control, with the following primers: 5′-TGAGCCCTTCCACAATGCCA-3′ and 5′-AGTGCCAGCCTCGTCCCGTA-3′. PCR products were analyzed using a 1.5% agarose gel. Gel images were digitally captured with a Gel Doc XR system (Bio-Rad, Hercules, CA, USA).

### 2.8. Measurement of Intracellular ROS Levels

For flow cytometric analysis, SV40 MES13 cells (1.5×10^5^ cells) were seeded on 60-mm dishes. The cells were incubated with vehicle (control) or 20 *μ*g/ml BKFE for 1 h and then further incubated with or without 100 *μ*M PA for 5 h. The cells were washed with DPBS. Next, 5 *μ*M of 2,7-dichlorodihydrofluorescein diacetate (DCFH-DA, Invitrogen, San Diego, CA, USA) was added, and the cells were incubated for 10 min at 37°C, harvested, and fixed with 5% neutral buffered formalin (NBF, Sigma, St. Louis, MO, USA) for 10 min at room temperature. Afterward, NBF was removed and the cells were resuspended in DPBS. DCF fluorescence intensity was detected by a FACS LSRII using the CellQuest™ Pro Software (BD Biosciences).

### 2.9. Immunocytochemistry

For ROS detection, SV40 MES13 cells (2.5×10^4^ cells/well) were cultured on coverslips in 24-well plates. The cells were incubated with vehicle (control) or 20 *μ*g/ml BKFE for 1 h and then further incubated with or without 100 *μ*M PA for 5 h. After changing the culture media, 10 *μ*M DCFH-DA (Invitrogen) was added and the cells were incubated for 30 min at 37°C. Fluorescence images were observed using a confocal microscope (LSM 700; Carl Zeiss Inc., Oberkochen, Germany). For Nrf2 detection, the cells (5×10^4^ cells/well) were cultured on coverslips in 12-well plates for 16 h, and then incubated with vehicle (control) or 1, 5, or 20 *μ*g/ml BKFE for a further 18 h. The coverslips were washed twice with DPBS and then fixed in 4% paraformaldehyde for 15 min at room temperature. The fixed cells were then washed with DPBS, blocked with DPBS containing 1% BSA and 0.1% Triton X-100 for 30 min at room temperature, and incubated overnight with anti-Nrf2 (Abcam) at 4°C. The cells were then stained with fluorescence-conjugated secondary antibody (Life Technologies, Carlsbad, CA, USA) for 2 h, stained with 4′,6-diamidino-2-phenylindole (DAPI, Invitrogen), mounted with VECTASHIELD® (Dako, Carpenteria, CA, USA), and then observed under a confocal microscope (Carl Zeiss Inc.). To evaluate Nrf2 expression, five random fields were selected in each experiment and 5-10 cells were imaged in each field. To quantitatively evaluate the fluorescent images, RGB images were analyzed by the ImageJ software and the mean value was used to obtain the bar graph.

### 2.10. Transfection

SV40 MES13 cells (1.2×10^5^ cells) were plated in 60-mm cell culture dishes without penicillin/streptomycin in culture medium for 16 h. Next, the cells were transfected with 75 pM of Nrf2 siRNA or scrambled siRNA (Santa Cruz Biotechnology) using Lipofectamine RNAiMAX reagent (Invitrogen) according to the manufacturer's instructions. After 11 or 24 h, the cells were incubated with vehicle (control) or 20 *μ*g/ml BKFE for 1 h and then further incubated with 100 *μ*M PA for 18 or 24 h.

### 2.11. Statistical Analysis

Statistical analysis was performed by the Student's t-test (two samples) or one-way ANOVA with Tukey's multiple comparison test (more than three samples) using the GraphPad Prism version 5 (GraphPad software Inc., San Diego, CA, USA). Data are presented as means ± standard error of the mean (SEM). Statistical significance was set at P < 0.05.

## 3. Results

### 3.1. BKFE Protects PA-Induced Lipotoxicity in SV40 MES13 Cells

**Figure 1:**
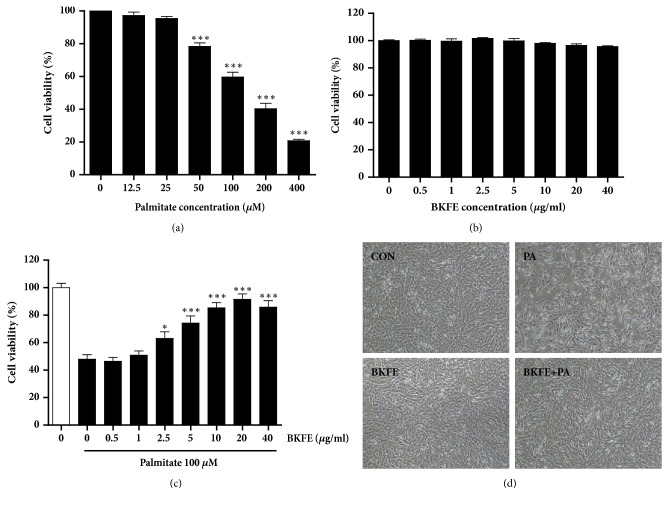
**BKFE protects PA-induced lipotoxicity in SV40 MES13 cells.** (a) SV40 MES13 cells were treated with the indicated concentrations of PA for 24 h. (b) Cells were treated with the indicated concentrations of BKFE for 24 h. (c) Cells were pretreated with 0.5-40 *μ*g/ml BKFE for 1 h, and then further incubated with 100 *μ*M PA for 24 h. Cell viability was determined by the CCK assay (three independent experiments). (d) Morphology of cells in the control (CON), 100 *μ*M palmitate-treated (PA), 20 *μ*g/ml BKFE-treated (BKFE), and 20 *μ*g/ml BKFE-pretreated (1 h) and 100 *μ*M palmitate-treated (24 h) (BKFE+PA) groups was examined by light microscopy (original magnification, ×40). *∗*p<0.05, *∗∗*p<0.01, and *∗∗∗*p<0.001.

We first examined the viability of SV40 MES13 cells treated with different concentrations of PA. SV40 MES13 cells were treated with 12.5-400 *μ*M PA for 24 h, and cell viability was measured by the CCK assay. As shown in Figure 1(a), cell viability was significantly decreased from 50 *μ*M PA in a dose-dependent manner compared with control cells (Figure 1(a)). We chose the concentration of 100 *μ*M PA, which showed a 59% cell viability, in the subsequent studies. In SV40 MES13 cells treated with 0.5-40 *μ*g/ml BKFE for 24 h, BKFE alone did not show any cytotoxicity (Figure 1(b)). To examine the effect of BKFE on PA-induced lipotoxicity, the cells were pretreated with BKFE (0.5-40 *μ*g/ml) for 1 h and then treated with 100 *μ*M PA. Pretreatment with 2.5-40 *μ*g/ml BKFE significantly increased cell viability in the presence of PA compared with no BKFE pretreatment. The protective effect of BKFE against lipotoxicity was most effective at 20 *μ*g/ml (Figure 1(c)). Changes in the cellular morphology clearly showed that PA treatment induced cytotoxicity; however, BKFE pretreatment inhibited this PA-induced cytotoxicity in SV40 MES13 cells (Figure 1(d)), indicating that BKFE had an ability to protect mesangial cells from PA-induced lipotoxicity.

### 3.2. BKFE Inhibits PA-Induced Apoptosis in SV40 MES13 Cells

**Figure 2:**
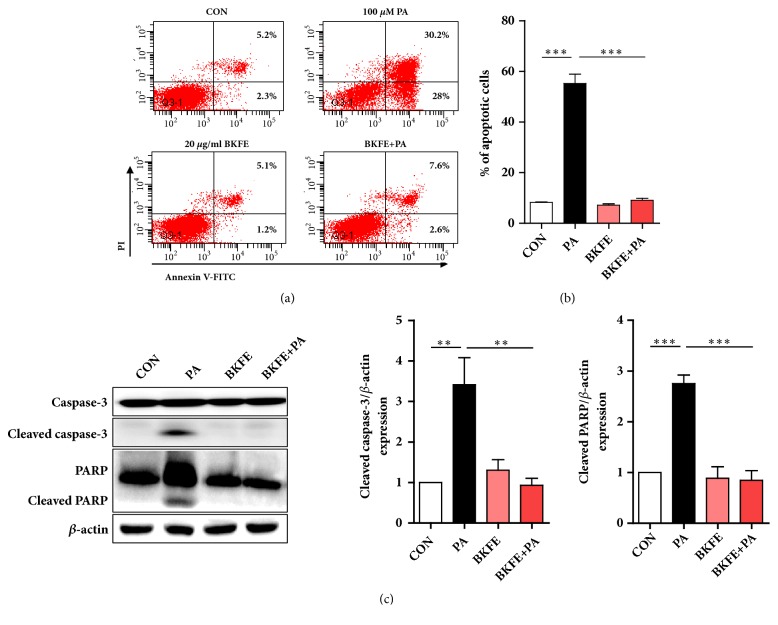
**BKFE inhibits PA-induced apoptosis in SV40 MES13 cells.** (a) SV40 MES13 cells were pretreated with 20 *μ*g/ml BKFE for 1 h and then further incubated with 100 *μ*M PA for 24 h. Cell apoptosis was analyzed by flow cytometry using the Annexin V (FITC)/PI binding assay. Percentage on representative flow cytometry data indicates the early (the lower right quadrant) and late (the upper right quadrant) apoptotic cells. (b) Percentage of apoptotic cells was calculated by the sum of early and late apoptotic cells. (c) SV40 MES13 cells were pretreated with 20 *μ*g/ml BKFE for 1 h and then further incubated with 100 *μ*M PA for 18 h. The protein levels of PARP and Caspase-3 were measured by western blotting (five independent experiments). The relative expression of the proteins was normalized to that of *β*-actin and quantified using the ImageJ software. *∗∗*p<0.01, and *∗∗∗*p<0.001.

To confirm the protective effect of BKFE on PA-induced lipotoxicity in mesangial cells, we determined the apoptosis of SV40 MES13 cells by flow cytometry using FITC-conjugated Annexin V and PI staining. PA significantly increased the population of Annexin V-FITC^+^ cells, whereas pretreatment with BKFE significantly inhibited PA-induced apoptosis (control, 8.2 ± 0.7%; PA, 58.9 ± 6.4%; BKFE, 7.0 ± 0.8%; BKFE+PA, 9.9 ± 1.3%; Figures 2(a) and 2(b)). We examined the expression of cleaved caspase-3 and cleaved Poly (ADP-Ribose) Polymerase (PARP) as apoptotic markers [6]. As expected, PA treatment increased the expression of these proapoptotic proteins in SV40 MES13 cells, whereas pretreatment with BKFE significantly inhibited this increase (Figure 2(c)). BKFE alone did not significantly change the expression levels of these proteins compared with those in the control cells.

### 3.3. BKFE Inhibits ER Stress in PA-Treated SV40 MES13 Cells

**Figure 3:**
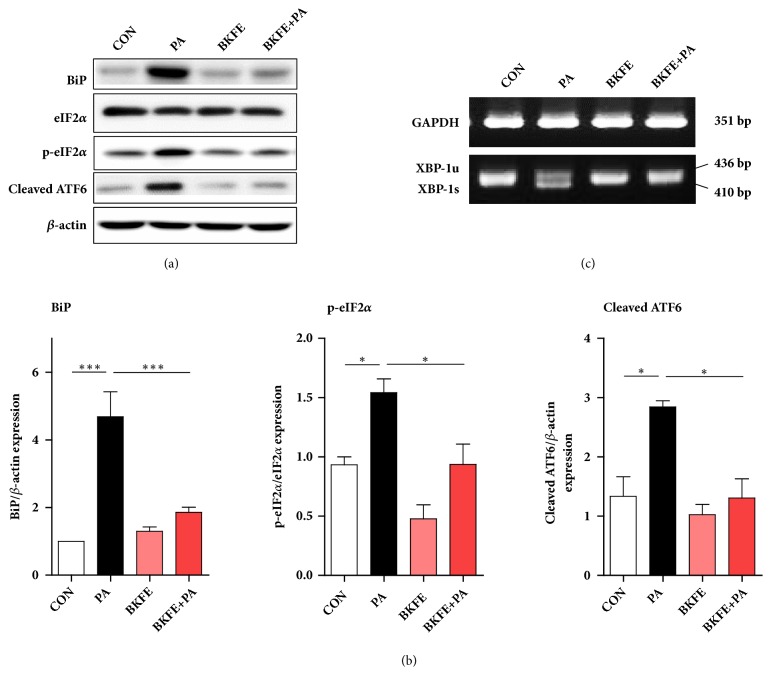
**BKFE inhibits ER stress in PA-treated SV40 MES13 cells.** (a) SV40 MES13 cells were pretreated with 20 *μ*g/ml BKFE for 1 h and then treated with 100 *μ*M PA for 18 h. The protein levels of ER stress-related genes were measured by western blotting (three to six independent experiments). (b) The relative expression of the proteins was normalized to that of *β*-actin and quantified using the ImageJ software. *∗*p<0.05, *∗∗*p<0.01, and *∗∗∗*p<0.001. (c) Cells were pretreated with 20 *μ*g/ml BKFE for 1 h and then treated with 100 *μ*M PA for 9 h. XBP-1 mRNA splicing was analyzed using RT-PCR (three independent experiments).

PA causes mesangial cell apoptosis by inducing ER stress [5]. ER stress signals are mediated by three major UPR sensors: inositol requiring element-1 (IRE-1)-splicing of X-box binding protein 1 (XBP-1), double-stranded RNA-activated protein kinase (PKR)-like ER kinase (PERK)-eIF2*α* activation, and activating transcription factor 6 (ATF6) [10]. To investigate which signaling pathways are involved in ER stress-induced mesangial cell death, we examined the expression of key signaling molecules in the UPR pathway. As shown in Figure 3, PA increased the expression of BiP, as well as the activation of eIF2*α* and ATF6. However, BKFE pretreatment significantly reduced the expression of these genes compared with that in PA-treated cells (Figures 3(a) and 3(b)). In addition, XBP-1 splicing in SV40 MES13 cells was increased by PA treatment, and this splicing was decreased by BKFE pretreatment (Figure 3(c)). These data showed that BKFE protected mouse mesangial cells from ER stress.

### 3.4. BKFE Inhibits ROS Production in PA-Treated SV40 MES13 Cells

**Figure 4:**
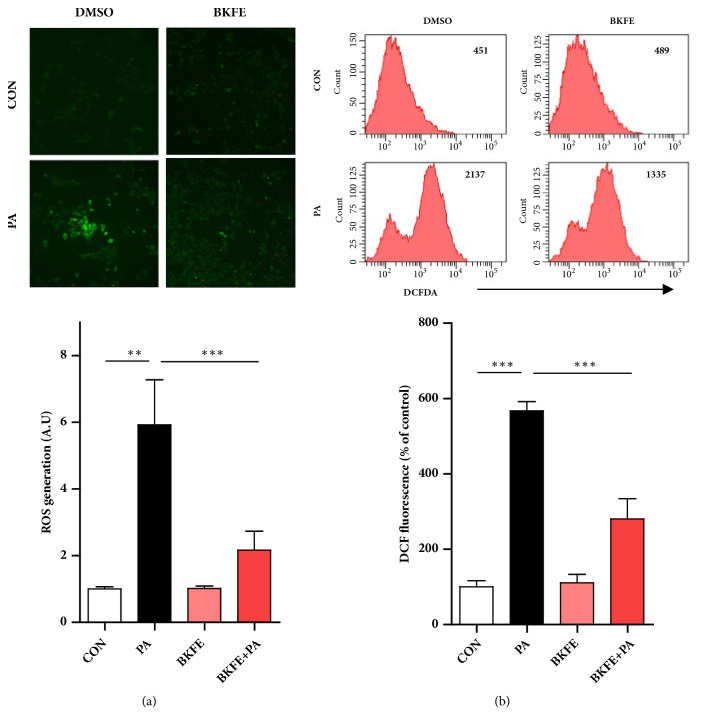
**BKFE inhibits ROS production in PA-treated SV40 MES13 cells.** SV40 MES13 cells were pretreated with 20 *μ*g/ml BKFE for 1 h and then treated with 100 *μ*M PA for 5 h. (a) Intracellular ROS levels were determined using confocal microscopy on cells stained with the ROS-sensitive fluorescent dye DCFH-DA (original magnification, ×100). Relative fluorescence level was quantified using the ImageJ software. (b) ROS production in the cells was measured by flow cytometry with DCFH-DA. Values on the representative flow cytometry data indicate the DCF fluorescence intensity of whole cells. Relative levels of DCF fluorescence intensity were compared with ROS production in the control. *∗∗*p<0.01, and *∗∗∗*p<0.001.

To determine whether the protective effect of BKFE on PA-induced ER stress and apoptosis is due to regulation of ROS production, we measured intracellular ROS level by observing DCF fluorescence intensity of cells treated with 100 *μ*M PA in the presence or absence of BKFE. As expected, ROS production in SV40 MES13 cells was significantly increased by PA treatment, however, pretreatment with BKFE significantly reduced PA-induced ROS production, as shown by the results of the microscopic (Figure 4(a)) and FACS analyses (Figure 4(b)).

### 3.5. BKFE Activates the Transcription Factor Nrf2

**Figure 5:**
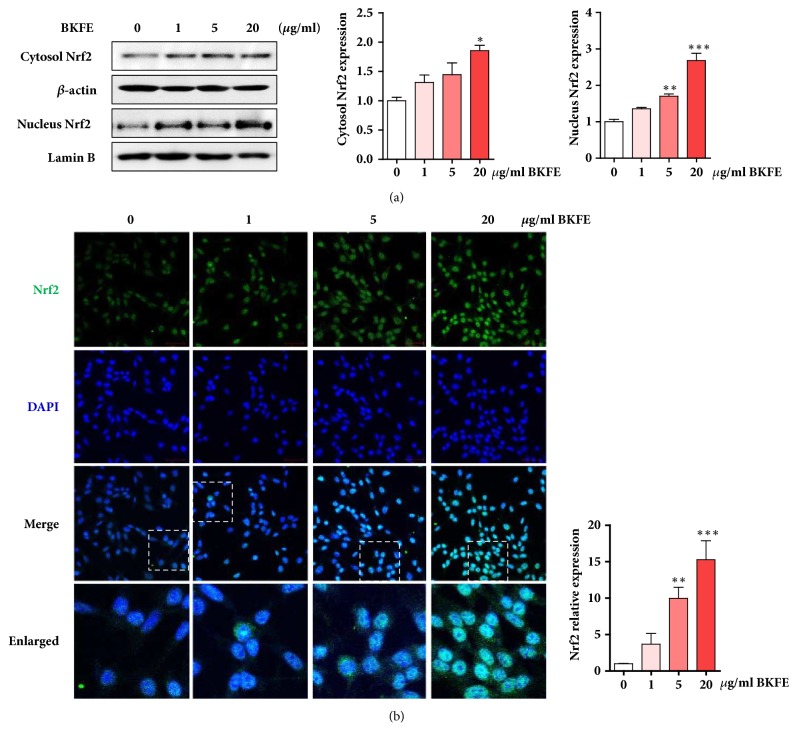
**BKFE activates the transcription factor Nrf2 in SV40 MES13 cells.** (a) SV40 MES13 cells were treated with 0, 1, 5, or 20 *μ*g/ml BKFE for 18 h. Cytoplasmic and nuclear Nrf2 proteins were extracted and analyzed by western blotting. Lamin B and *β*-actin were used as loading controls for the nuclear and cytosolic fractions, respectively. The relative expression of Nrf2 was quantified using the ImageJ software. (b) Cells were seeded on coverslips and treated with 0, 1, 5, or 20 *μ*g/ml BKFE for 18 h. Immunocytochemistry was performed using an anti-Nrf2 antibody and FITC-conjugated secondary antibody. Cell nuclei were counterstained with DAPI (blue). Boxed areas are enlarged. Magnifications, ×200 and ×850 (boxed areas). The relative fluorescence expression of Nrf2 was quantified using the ImageJ software. *∗*p<0.05, *∗∗*p<0.01, and *∗∗∗*p<0.001.

The transcription factor nuclear factor (erythroid-derived 2)-like 2 (Nrf2) is well-known as a master regulator of antioxidants and phase-II detoxification enzymes [22]. In addition, Nrf2 is translocated into the nucleus under oxidative stress. To examine BKFE-induced changes in the cellular localization of Nrf2, cytosolic and nuclear proteins were extracted from BKFE-treated SV40 MES13 cells. The results of western blotting analysis showed that both the cytosolic and nuclear levels of Nrf2 were increased in a dose-dependent manner in BKFE-treated cells (Figure 5(a)). By immunofluorescence staining, we confirmed that BKFE increased the nuclear translocation of Nrf2 in a dose-dependent manner (Figure 5(b)).

### 3.6. BKFE Induces the Expression of Antioxidant-Related Genes via Activation of Nrf2 Signaling

**Figure 6:**
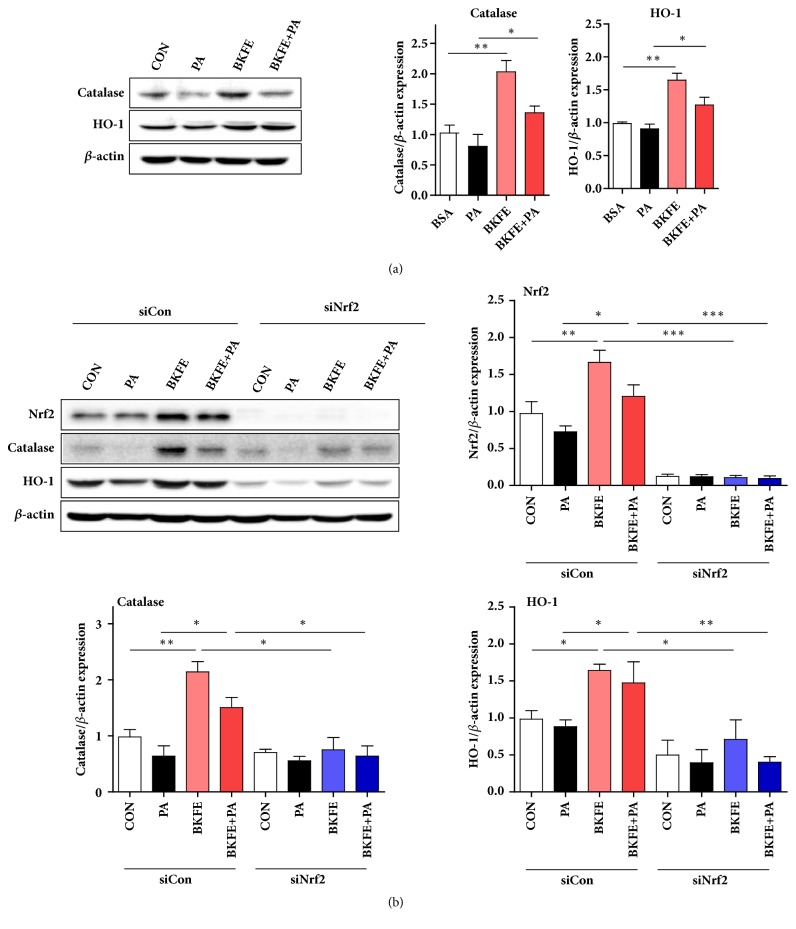
**BKFE induces antioxidant-related genes expression via Nrf2 signaling.** (a) SV40 MES13 cells were pretreated with vehicle or 20 *μ*g/ml BKFE for 1 h and then treated with 100 *μ*M PA for 18 h. The protein levels of antioxidant-related genes were analyzed by western blotting. The relative expression of the proteins was normalized to that of *β*-actin and quantified using the ImageJ software (three independent experiments). (b) Cells were transfected with scrambled siRNA (siCon) or Nrf2 siRNA (siNrf2) for 11 h. After incubation, the cells were pretreated with vehicle or 20 *μ*g/ml BKFE for 1 h, and then treated with 100 *μ*M PA for 18 h. The protein levels of Nrf2, and antioxidant-related genes were measured by western blotting. The relative expression of the proteins was normalized to that of *β*-actin and quantified using the ImageJ software (three independent experiments). *∗*p<0.05, *∗∗*p<0.01, and *∗∗∗*p<0.001.

To investigate whether BKFE-induced activation of Nrf2 increases the expression of Nrf2-target genes, we examined the expression of antioxidant-related factors in SV40 MES13 cells treated with 100 *μ*M PA in the absence or presence of BKFE. Compared with that in untreated control cells, the expression of catalase and HO-1 was significantly increased by BKFE treatment, and their expression, compared with that in PA-treated cells, was significantly increased by BKFE pretreatment (Figure 6(a)). To further confirm whether Nrf2 truly plays a role in BKFE-induced increase in catalase and HO-1 levels, we examined the expression of these genes in Nrf2 siRNA-transfected SV40 MES13 cells treated with 100 *μ*M PA in the absence or presence of BKFE. Nrf2 expression was significantly reduced after 30 h of Nrf2 siRNA transfection (Figure 6(b)). As expected, the expression of catalase and HO-1 in scrambled siRNA-transfected cells was higher in the BKFE+PA treatment group than in the PA treatment group; however, this increase was significantly reduced in Nrf2 siRNA-transfected cells (Figure 6(b)).

### 3.7. Knockdown of Nrf2 in SV40 MES13 Cells Reduced the Protective Effect of BKFE on PA-Induced Apoptosis

**Figure 7:**
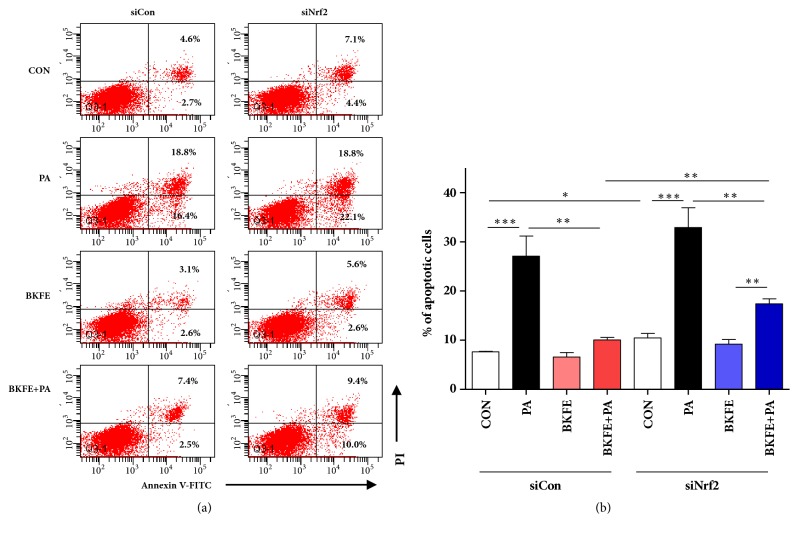
**BKFE protects against PA-induced apoptosis in SV40 MES13 cells via Nrf2 signaling.** (a) SV40 MES13 cells were transfected with siCon or siNrf2 for 24 h. The transfected cells were pretreated with or without 20 *μ*g/ml BKFE for 1 h and then treated with or without 100 *μ*M PA for 24 h. Cell apoptosis was analyzed by flow cytometry using the Annexin V (FITC)/PI binding assay. Percentage on the representative flow cytometry data indicates the early (the lower right quadrant) and late (the upper right quadrant) apoptotic cells. (b) Percentage of apoptotic cells was calculated by the sum of early and late apoptotic cells (three independent experiments). *∗*p<0.05, *∗∗*p<0.01, and *∗∗∗*p<0.001.

Finally, to confirm the protective effect of BKFE on PA-induced lipotoxicity via Nrf2 signaling, we determined the apoptosis of siNrf2-transfected SV40 MES13 cells by flow cytometry. Consistent with the results shown in Figure 2, pretreatment with BKFE significantly inhibited PA-induced apoptosis in control siRNA-transfected cells (Figures 7(a) and 7(b)). The inhibition of PA-induced apoptosis by BKFE was significantly reduced in siNrf2-transfected cells compared to that in control cells. However, the inhibitory effect of BKFE on PA-induced apoptosis was not completely abolished in siNrf2-transfected cells (Figures 7(a) and 7(b)).

## 4. Discussion

Natural plants have been used as alternative treatments for kidney diseases and their complications [12–14]. Japanese paper mulberry has been used in oriental medicine, and exhibits various pharmacological activities. The leaf of BK inhibits atopic dermatitis-like responses [17]. The root bark of BK exerts an anticancer effect via inhibition of angiogenesis [16] or induction of ROS generation [19]. In addition, the root bark of BK exerts an anti-inflammatory effect through inhibition of NO production [18]. Interestingly, an extract of BK stem bark shows antidiabetic and antihyperglycemic activities in diabetic rats [23]. Moreover, its alkaloids have been reported as inhibitors of glycosidase, implying their potential as therapeutics for diabetes [24]. In the present study, we investigated the protective effects of BKFE on PA-induced toxicity in mesangial cells.

Increased FFA level, alone or with hyperglycemia, have been shown to trigger kidney damage [8, 9]. In addition, lipotoxicity induced by prolonged elevation in FFA levels, especially saturated FFAs, such as PA, leads to the apoptosis of kidney cells, including podocytes and mesangial cells [5, 6, 9]. In agreement with those of previous studies, our results showed that exposure to PA significantly induced cell death in mesangial cells. However, treatment with BKFE prevented PA-induced mesangial cell death. As shown in Figure 2, PA induced the expression of cleaved caspase-3 and cleaved PARP, as previously reported [6, 7], and pretreatment with BKFE suppressed the expression of these molecules, suggesting that BKFE exerted antiapoptotic effects in cells under lipotoxic conditions. It was reported that phenolic compounds from the root bark of BK also showed a protective effect in pancreatic *β*-cells against cytokine-induced toxicity via attenuation of apoptosis [25]. At present, we do not know whether active components in the root and fruit of BK are similar or not. Further studies are required to identify the active components of BKFE.

PA-induced apoptosis has been implicated in increased ER stress and intracellular ROS production in multiple cell types, including mesangial cells [4, 9, 21]. Furthermore, elevated cellular ROS level is linked with ER stress [26]. ER stress activates UPR, a complex signaling program mediated by three ER transmembrane receptors: ATF6, IRE1, and PKR-like PERK, and its major function is apoptosis [27, 28]. In the current study, we found that PA-induced elevation of phosphorylated eIF2*α*, cleaved ATF6, and spliced XBP-1 in SV40 MES13 cells were reversed by BKFE pretreatment. Further studies are needed to elucidate the signaling pathway, but these results indicated that BKFE can inhibit all three UPR signaling. Because the inhibition of the expression of ER stress-related genes inhibits ER stress-induced apoptosis in mesangial cells [29, 30], we suggest that pretreatment with BKFE suppressed apoptosis by inhibiting PA-induced ER stress.

ROS overproduction induces damage of cellular DNA and protein, resulting in cellular dysfunction and apoptosis [21, 31–33]. Excessive ROS causes oxidative stress and contributes to pathological conditions, such as diabetes, diabetic complications, cancer, and neurological disorders [34–36]. A key protective mechanism against oxidative stress is mediated via the expression of antioxidant genes. The transcription factor Nrf2 plays a major role in the regulation of redox homeostasis and induces the expression of cytoprotective and detoxification genes [37, 38]. In the present study, BKFE induced Nrf2 activation and upregulated the expression of Nrf2-target antioxidant enzymes, catalase and HO-1. Previous reports showed that the induction of the Nrf2/HO-1 antioxidant axis inhibits oxidative stress in mesangial cells, which results in cytoprotective effects [39–41]. Therefore, the induction of Nrf2 activation and HO-1 expression by BKFE might contribute in protecting SV40 MES13 cells from PA-induced toxicity. However, knockdown of Nrf2 did not completely abolish the expression of antioxidant enzymes or the antiapoptotic effects of BKFE, even though the expression of Nrf2 was completely reduced compared to the control cells. These results indicated that the protective effect of BKFE against PA-induced lipotoxicity was not solely due to Nrf2-dependent signaling. In fact, Nrf2-independent signaling might also be involved in the protective effect of BKFE against PA-induced lipotoxicity.

In conclusion, our study showed that BKFE can effectively protect SV40 MES13 cells from PA-induced lipotoxicity. Moreover, BKFE attenuated PA-induced ROS overproduction and ER stress in SV40 MES13 cells by elevating Nrf2 activation. Thus, BKFE could be used as a potential therapeutic agent to prevent mesangial cell damage caused by oxidative stress or lipotoxicity.

## Data Availability

No data were used to support this study.
